# Hyaluronan Synthase Isoforms Direct Distinct Patterns of Fibroblast Activation Linked to Fibrosis Progression Versus Resolution Following Renal Ischaemia

**DOI:** 10.3390/biom16101442

**Published:** 2026-10-03

**Authors:** Irina V. Grigorieva, Adam C. Midgley, Charlotte V. M. Brown, Gilda Pino Chavez, Barbara Szomolay, Aled Williams, Rachel DePaul, Richard J. Stanton, John M. Hancock, Timothy Bowen, Deva Chan, Anna Plaas, Rafael Chavez, Robert Steadman, Usman Khalid, Soma Meran

**Affiliations:** 1 Division of Infection and Immunity, School of Medicine, Cardiff University, Cardiff CF14 4XW, UK; grigorievai@cardiff.ac.uk (I.V.G.); charlotte.brown123@nhs.net (C.V.M.B.); gildapinochavez@gmail.com (G.P.C.); szomolayb@cardiff.ac.uk (B.S.); aled.williams6@wales.nhs.uk (A.W.); stantonrj@cardiff.ac.uk (R.J.S.); bowent@cardiff.ac.uk (T.B.); rafael.chavez@wales.nhs.uk (R.C.); khalidu@cardiff.ac.uk (U.K.); 2 Key Laboratory of Bioactive Materials, Ministry of Education, College of Life Sciences, Nankai University, Tianjin 300071, China; midgleyac@nankai.edu.cn; 3 Institute of Biochemistry and Molecular Genetics, Faculty of Medicine, University of Ljubljana, Zaloska 4, 1000 Ljubljana, Slovenia; jmhancock@gmail.com; 4 Weldon School of Biomedical Engineering, Purdue University, West Lafayette, IN 47907, USA; chand@purdue.edu; 5 Department of Internal Medicine, Division of Rheumatology, Rush Medical College, Rush University, Chicago, IL 60612, USA; aplaas@gmail.com; 6 Cardiff Transplant Unit, Cardiff and Vale University Hospital of Wales, Cardiff CF14 4XW, UK

**Keywords:** hyaluronan, HAS enzymes, fibrosis, stroma

## Abstract

The stroma plays key roles in development and tissue regeneration after injury, but its expansion following injury can drive fibrosis and chronic disease. In the kidney, hyaluronan (HA) is largely absent from the healthy stroma but becomes abundant during disease. To investigate HA regulation in stromal responses, we compared HA and hyaluronan synthase (HAS) expression during kidney development, homeostasis, injury, and recovery. A rat model of ischaemia–reperfusion injury (IRI) with or without ischaemic pre-conditioning (IPC) was studied alongside embryonic and *Has1/3*^−/−^ mouse kidneys, with mechanistic studies performed in primary human fibroblasts. In healthy kidneys, stromal HA was absent, HAS1 showed limited epithelial expression, and HAS2 was undetectable. Following IRI, stromal HA accumulation and increased HAS2 expression were associated with chronic fibrosis. In contrast, during development, prominent stromal HA matrices were evident in areas of tubular generation, with strong HAS1 (not HAS2) expression. During recovery, stromal HA and HAS2 were attenuated, whereas HAS1+ stromal cells expanded but were distinct from α-SMA+ myofibroblasts. Functionally, HAS1+ fibroblasts displayed enhanced migration, fibroblast activation protein (FAP) expression and reduced profibrotic marker expression, whereas HAS2+ fibroblasts exhibited a contractile myofibroblast phenotype with high extra domain A fibronectin (EDA-FN) and type I collagen, alpha-1 subunit (COL1A1). Together, these findings identify HA as a regulator of stromal fibroblast phenotype and associate HAS1 and HAS2 in divergent fibrotic outcomes following kidney injury.

## 1. Introduction

The past decade has seen an increased appreciation of the high prevalence, morbidity and mortality of acute kidney injury (AKI) and chronic kidney disease (CKD) in developing as well as developed populations. Furthermore, AKI and CKD are not two distinct entities, with even minor AKI leading to CKD through the process of progressive fibrosis [1,2,3,4].

Ischaemia–reperfusion injury (IRI) is the leading cause of AKI [5,6]. IRI describes the tissue damage that occurs when the blood supply to an organ is interrupted, followed by re-instatement of blood flow and subsequent re-oxygenation. Ischaemia results in tissue hypoxia, and reperfusion augments tissue injury through activation of immune, inflammatory, and apoptotic pathways [7,8]. Together, sustained activation of these pathways drives progressive fibrosis resulting in organ damage and CKD.

In clinical settings, IRI develops in the context of disease processes linked with poor renal perfusion such as sepsis, heart disease and shock. It affects between 10 and 15% of hospitalized patients and is associated with poor patient outcomes [3,8,9]. Identifying mechanisms that can alleviate kidney damage associated with IRI is therefore key to reducing the burden of kidney disease, and to understanding pathways that drive progressive kidney fibrosis. Moreover, it may be possible to exploit these mechanisms to address fibrosis in other organs.

Fibrosis is the principal pathological process that underlies all progressive renal disease. It results from an imbalance between extracellular matrix (ECM) deposition and degradation leading to excessive fibrous scar accumulation and renal parenchymal damage. α-smooth muscle actin positive (α-SMA+) myofibroblasts are the principal effector cells that drive fibrosis and are derived from differentiation of stromal cells including resident fibroblasts and pericytes under the influence of pro-fibrotic cytokines, such as Transforming Growth Factor-β1 (TGF-β1) [10,11,12,13,14,15].

Fibroblasts have a common stromal progenitor cell-lineage but display considerable phenotypic plasticity. Indeed, recent advances in lineage-tracing and single-cell RNA sequencing have confirmed the existence of distinct sub-populations of renal fibroblasts that are transcriptionally, functionally, and spatially heterogeneous [16,17,18]. However, it has been difficult to enrich and isolate these subpopulations from kidneys sufficiently to understand their biology.

These stromal cells are key players in maintaining homeostasis and in facilitating repair and regeneration. In renal development they are critical for differentiation and maturation of nephrons. In the regenerative field, stromal cells assembled in co-culture with nephron and ureteric bud progenitors lead to the formation of organoids that closely resemble organotypic renal architecture and function, including the formation of nephron tubules [19]. By contrast, maladaptive fibroblast responses drive fibrosis and progressive CKD.

Despite the increasing worldwide prevalence of renal disease, there is a limited understanding of fibroblast heterogeneity in the kidney and the factors that regulate phenotypic variations. Therefore, insight into the distinct differences in fibroblast subpopulations during embryonic development and health, and how these can be recapitulated to promote renal recovery following injury, is crucial to further advances in this field.

Oral mucosal and dermal fibroblasts provide a well-established model of regenerative and scar-forming fibroblast phenotypes respectively. Oral mucosal fibroblasts heal with minimal scarring, whereas dermal fibroblasts display a profibrotic phenotype that recapitulates key biological features of fibroblasts in fibrotic organs including the kidney, lung and liver. Importantly, donor-matched oral and dermal fibroblasts isolated from the same individual minimize inter-individual variability, providing a robust system in which to investigate the molecular mechanisms that drive the phenotypic switch from pro-fibrotic to pro-regenerative fibroblasts.

Hyaluronan (HA) is an ECM glycosaminoglycan that is critical in kidney development and demonstrates increased expression in the renal stroma during progressive kidney disease [20,21,22]. Our previous cell studies indicate that distinct alterations in pericellular HA generation, assembly and interactions are key to determining fibroblast heterogeneity [23,24,25,26,27].

HA is synthesized at the cell membrane by three HA Synthase (HAS) isoenzymes, HAS1, HAS2 and HAS3. The genes encoding these isoenzymes are located at discrete genomic loci, and cross-species gene- and protein-sequence conservation underlines their key roles in a variety of biological functions. In this study we show that HAS1 and HAS2 drive distinct fibroblast phenotypes in vitro and are identified in distinct subpopulations of renal stromal cells in vivo linked with fibrosis progression versus resolution following IRI. The results provide novel insights into HAS isoenzymes as distinct regulators of stromal responses in progressive renal injury.

## 2. Materials and Methods

### 2.1. Animals

Experiments were performed in line with institutional and UK Home Office guidelines under the authority of an appropriate project licence. Adult male Lewis rats (8 to 12 weeks) were supplied by Charles River Laboratories (Margate, Kent, UK). All experimental procedures were carried out in accordance with local policies at JBIOS, Cardiff University and licenced under the Animal (Scientific Procedures) Act 1986, issued by the UK Government Home Office Department. Rats were housed in conventional housing with readily available chow and water. One day (24 h) prior to surgery, Buprenorphine (Temgesic) was added to drinking water to provide analgesia. Isoflurane anesthesia was delivered via an induction chamber. The ARRIVE guidelines were followed for all in vivo experiments. Based on previous work, a power calculation indicated *n* = 8 per group to give adequate power (alpha 0.05, beta 0.8).

IRI model—The central abdomen was shaved and a midline laparotomy incision made. Bilateral IRI was performed by clamping both renal pedicles for 45 min and kidneys visually assessed for signs of ischaemia. The clamps were then removed, wound closure performed and animals recovered [28,29]. Sham animals underwent a midline laparotomy alone with no renal pedicle clamping. Kidney tissues were retrieved under terminal anesthesia at 2 days (48 h), 7 days, 14 days and 28 days post-operatively. Blood samples for serum creatinine were taken at the same times. Kidneys were divided longitudinally with one half stored in formalin for histological evaluation and one half placed in RNALater (Thermofisher Scientific, Waltham, MA, USA). A minimum of 8 rats were used per group according to appropriate power calculations.

IPC model—A group of rats underwent ischaemic pre-conditioning (IPC) prior to IRI. In this group, bilateral renal pedicles were clamped for 2 min (ischaemia), followed by release of clamps for 5 min (reperfusion) [28,29,30]. This process was performed three times in total, prior to bilateral IRI (cross-clamping of both renal pedicles for 45 min). A group of animals underwent IPC alone with no subsequent IRI to investigate the effects of IPC alone. Kidneys tissues and blood samples were retrieved as above at 2, 7, 14 and 28 days post-operatively. A minimum of 8 rats were used per group according to appropriate power calculations.

*Has1*^−/−^*Has3*^−/−^ and wild-type Mice—Mouse kidneys from wild-type (WT) and *Has1* and *Has3* double-knockout (KO) male C57BL/6 mice were supplied by Professor Anna Plaas (Rush University, Chicago, IL, USA) and approved according to Rush University IACUC. Breeding, experimental procedures and maintenance were undertaken as previously described [31]. Mice had free access to water and chow and were exposed to the same light cycles (12 h light, 12 h dark) during the growth period. Mice were sacrificed at 12 weeks of age by CO_2_ asphyxiation and cervical dislocation. Kidneys were harvested and placed in formalin or RNALater for histological or gene-expression analysis.

Embryonic Studies—Embryos were collected with the day of the vaginal plug designated as embryonic (E) day 0.5 and staged by morphologic criteria, which included somite number and eye and limb morphology. Kidneys were dissected from embryonic (E) day 15.5 embryos, a time at which all progenitor populations and maturing cell types co-exist, using a stereomicroscope.

### 2.2. Primary Human Cells

Scarring fibroblast (SF) and non-scarring fibroblast (NSF) phenotypes—Donor-matched samples of dermal (scarring) and oral mucosal (non-scarring) fibroblasts (OMFs) were obtained by biopsy from consenting adults undergoing routine minor surgery (ethical approval obtained from South-East Wales Research Ethics Committee, Reference No. 02/4836, 2002). The cells were isolated as previously described [32]. OMFs are intrinsically regenerative and heal with minimal scarring, whereas dermal fibroblasts display a profibrotic phenotype that shares key biological features with fibroblasts from fibrotic organs, including the kidney, lung and liver. We have previously established this donor-matched system as a model to investigate mechanisms regulating the fibroblast switch from SF to NSF phenotypes.

Cell Culture—The above cells were cultured in Dulbecco’s modified Eagle’s medium and F-12 medium containing 2 mM L-glutamine, 100 units/mL penicillin, and 100 μg/mL streptomycin supplemented with 10% fetal bovine serum (FBS) (Biologic Industries Ltd., Cumbernauld, UK). The cells were maintained at 37 °C in a humidified incubator in an atmosphere of 5% CO_2_, and fresh growth medium was added to the cells every 3–4 days until confluent. The cells were incubated in serum-free medium for 48 h before use in experiments, and all experiments were done under serum-free conditions unless otherwise stated. All experiments were undertaken using cells at passage 6–10. Experimental conditions were designed such that cells were either incubated in serum-free medium (control) or in medium containing 10 ng/mL TGF-β1 for 72 h.

Construction of Adenoviral Vectors and NSF Infection—Adenoviral vectors for enforced expression of *HAS1* and *HAS2* open-reading frames (ORFs) were constructed by PCR amplification of the *HAS* ORFs from the plasmid expression vectors described above, followed by homologous recombination into the replication deficient, E1/E3 deleted, adenoviral vector pAdZ-CV5 as described elsewhere [33]. OMFs were grown to 70% confluence and growth arrested for 24 h in serum-free medium. Viral vectors were added at multiplicity of infection (MOI) of 40 PFU/cell to the cells in a minimal volume of serum-free medium and rocked for 2 h before replacement with serum-free medium for 48 h and subsequent experimentation with serum-free medium alone or containing 10 ng/mL TGF-β1.

siRNA Knockdown of Gene Expression—Annealed oligonucleotide siRNA reagents for the knockdown of *HAS1* (assay ID 119443), *HAS2* (s6458), and scrambled negative control transfection (4611) were purchased from Life Technologies (Carlsbard, CA, USA) and used in accordance with the manufacturer’s guidelines. A final concentration of 30 nM of each siRNA was transfected into cultured cells using Lipofectamine 2000 (Life Technologies, Carlsbad, CA, USA), as described previously [23].

### 2.3. Reverse Transcription (RT) and Quantitative PCR (qPCR)

qPCR was used to quantify mRNA expression levels of *HAS1*, *HAS2*, *CDH1* (E-Cadherin), *ACTA2* (α-SMA), *HYAL2* (hyaluronidase 2), *FN1* (Fibronectin), EDA-FN (extra domain-A–fibronectin), *COL1A1* (collagen type I alpha 1 chain) and *FAP* (Fibroblast Activated Protein). Total RNA was extracted from frozen kidney tissue stored in RNALater with RNeasy mini kit (Qiagen, Hilden, Germany), quantified by NanoDrop spectrophotometer and used to prepare cDNA using the Multiscribe reverse transcriptase (Thermo Fisher Scientific, Waltham, MA, USA). qPCR was performed using Power SYBR Green or Taqman PCR Master Mix (Thermo Fisher Scientific, Waltham, MA, USA) and specific oligonucleotide primers. Samples were analyzed on a ViiA 7 Real-Time qPCR System (Thermo Fisher Scientific, Waltham, MA, USA), and relative expression levels were normalized to a standard reference gene (18S rRNA) using the 2^−ΔΔCt^ method [23].

### 2.4. Histology, Immunohistochemistry (IHC) and Immunofluorescence (IF)

Fixed rat or mouse kidneys were processed for embedding in paraffin and cut into 5 μm sections for Masson’s Trichrome staining, HA-binding protein (HABP) staining or immunostaining. Deparaffinized kidney sections were rehydrated in graded alcohols (100%, 96%, 70%, and 50%), and antigen retrieval was performed in sodium citrate buffer in the autoclave at 120 °C for 20 min. For IHC signal detection, endogenous peroxidase activity was quenched by incubation in 3% (*v*/*v* H2O2/methanol for 10 min at room temperature. Where biotinylated secondary antibodies were used, sections were incubated with avidin/Biotin block (Vector Laboratories, Newark, CA, USA) for 10 min. Sections were blocked in 10% goat serum (Vector Laboratories, Newark, CA, USA) for 20 min at room temperature and incubated overnight at 4 °C with primary antibodies. Immobilized antibodies were detected by UltraVision-LP HRP Polymer detection system specific for anti-mouse and anti-rabbit IgG (ThermoFisher Scientific, Waltham, MA, USA), or with biotinylated secondary antibodies and AB reagents (Vector Laboratories, Newark, CA, USA) according to manufacturer’s instructions. DAB (Vector Laboratories, Newark, CA, USA) and hematoxylin were used as the chromogen and the nuclear counterstain, respectively. For detection of HA, a HA-binding protein (HABP, Merck, Darmstadt, Germany; Ref: 385911) with a biotin conjugate was used (1:200) followed by an AB reagent or streptavidin conjugated with AlexaFlour 488/594 fluorophore (ThermoFisher Scientific, Waltham MA, USA). For IF detection, AlexaFluor 488 goat anti-mouse IgG (H + L) and Alexa Fluor 594 goat anti-mouse IgG (H + L) secondary antibodies were used at 1:500 dilution (Thermo Fisher Scientific, Waltham, MA, USA). DAPI (4′,6-diamidino-2-phenylindole) was used to visualize the nuclei. In negative controls, primary antibodies or HABP were omitted. Brightfield kidney sections were examined using a Leica DMLA Light Microscope (Leica Microsystems, Wetzlar, Germany) equipped with an Olympus DP27 Microscope digital camera (Olympus Corporation, Tokyo, Japan). For IF, kidney sections were examined and imaged using a Zeiss LSM880 Airyscan Confocal Laser Scanning Microscope (Carl Zeiss Microscopy GmbH, Jena, Germany). Histological assessment was performed independently by a blinded renal histopathologist. Left and right kidneys were assessed separately according to the predefined histological scoring criteria [29]. For immunofluorescence analyses, tissue from a minimum of three independent biological replicates was examined. Both left and right kidneys were analyzed, with a minimum of ten representative fields of view assessed per kidney to account for tissue heterogeneity. The biological replicate (individual animal) was the experimental unit for all in vivo analyses.

### 2.5. Visualization of Pericellular HA by Particle-Exclusion Assay

Pericellular HA coats were visualized by the exclusion of horse erythrocytes as reported previously [26]. Formalized horse erythrocytes were washed in PBS and centrifuged at 1000× *g* for 7 min at 4 °C. The pellet was resuspended in serum-free medium at an approximate density of 1 × 10^8^ erythrocytes/mL. A total volume of 500 μL of this suspension was added to each 35 mm dish containing sub-confluent cells and swirled gently for even distribution. The dishes were incubated at 37 °C for 15 min to allow the erythrocytes to settle around the cells. Control cells were incubated with 200 μg/mL bovine testicular hyaluronidase in serum-free medium for 30 min prior to the addition of formalized horse erythrocytes. On settling, the erythrocytes were excluded from zones around the cells with HA pericellular coats. This was viewed under the microscope as an area of erythrocyte exclusion. Zones of exclusion were visualized on a Zeiss Axiovert 135 inverted microscope (Carl Zeiss, Oberkochen, Germany).

### 2.6. Collagen Gel Contractility Assay

Type I collagen was extracted from rat-tail tendon as previously described [34]. Approximately 2.5 × 10^5^ fibroblasts/mL were mixed into collagen lattice-forming solutions (2.5 mL 20% *v*/*v* fetal calf serum–Dulbecco’s modified Eagle’s low-glucose medium, 500 μL of 0.1 mol/L NaOH, and 2 mg/mL type I collagen; total volume of 5 mL). Fibroblast-populated collagen lattices (FPCLs) were maintained at 37 °C, in a 5% CO_2_ atmosphere for 1 h, for collagen polymerization to occur. FPCLs were gently detached from the plate edges and resuspended in serum-free medium containing appropriate treatments. FPCLs were measured at 0, 24, 48, 72, 96, 120, and 144 h after initial lattice fabrication. The mean FPCL contraction values were obtained from analysis by ImageJ software (version 1.37c; National Institute of Health, Bethesda, MD, USA).

### 2.7. Scratch-Wound Migration Assay

Scratching quiescent fibroblasts cell monolayers with sterile 200-μL pipette tips generated linear denuded areas. The cells were gently washed with PBS to remove detached cells and then replenished with fresh serum-free medium containing appropriate treatments. The wound width was photographed at 0, 24, 48, and 72 h or until closure, using an Axiovert 100 inverted microscope (Carl Zeiss, Oberkochen, Germany) fitted with a digital camera (Orca-1394; Hamamatsu Photonics K.K., Hamamatsu, Japan). Measurements were obtained using ImageJ software version 1.37c and the area of wound closure was measured to assess the rate of migration.

### 2.8. In Silico Sequence Analysis

A region totalling 3 kb upstream of the HAS isoforms’ transcription start sites was extracted from the Ensembl genome browser (https://www.ensembl.org/), taking sequence from position −1 to −3000 with respect to the transcription start sites, and was analyzed for conserved noncoding regions (CNRs) using VISTA [35].

### 2.9. Statistical Analysis

Animal numbers were determined a priori based on previous studies and power calculations, which indicated that *n* = 8 animals per group would provide adequate statistical power (α = 0.05, β = 0.8 (80% power)), whilst minimizing animal use in accordance with the ARRIVE guidelines and the principles of the 3Rs. The experimental cohort sizes were as follows: Sham: 24 h (*n* = 8), 48 h (*n* = 11), 7 d (*n* = 4), 14 d (*n* = 4) and 28 d (*n* = 8); IRI: 24 h (*n* = 8), 48 h (*n* = 13), 7 d (*n* = 4), 14 d (*n* = 4) and 28 d (*n* = 8); IPC + IRI: 24 h (*n* = 8), 7 d (*n* = 4), 14 d (*n* = 4) and 28 d (*n* = 8).

All in vitro experiments were performed as three independent biological experiments using fibroblasts isolated from three separate donor-matched oral mucosal and dermal fibroblast pairs. Each biological experiment included appropriate technical replicates, and qPCR analyses were performed using technical replicate measurements. Statistical analyses were performed using the biological replicate as the experimental unit. For in vivo analyses, the individual animal constituted the biological replicate and experimental unit. Measurements obtained from multiple microscopic fields of view and tissue sections were used to provide a representative assessment of each animal and were not treated as independent biological observations for statistical analysis. Where both kidneys from the same animal were analyzed, they were not considered independent biological replicates.

Data were assessed for normality and homogeneity of variance using GraphPad Prism prior to statistical analysis. Parametric tests were applied only where these assumptions were satisfied. The two-tailed unpaired *t*-test was used to assess statistical differences between two experimental groups. For experiments with multiple experimental groups, one-way analysis of variance was used to identify statistical differences across groups, followed by post-test Bartlett and multiple comparisons. For experiments with multiple experimental conditions, two-way analysis of variance was used, followed by post-test Tukey multiple comparisons. Graphical data are expressed as means ± standard error of mean (SEM). All data were analyzed using GraphPad Prism software version 6 (GraphPad Software, San Diego, CA, USA). *p* < 0.05 was considered statistically significant.

## 3. Results

### 3.1. HA Distribution in Uninjured Adult Rat Kidneys

**Figure 1:**
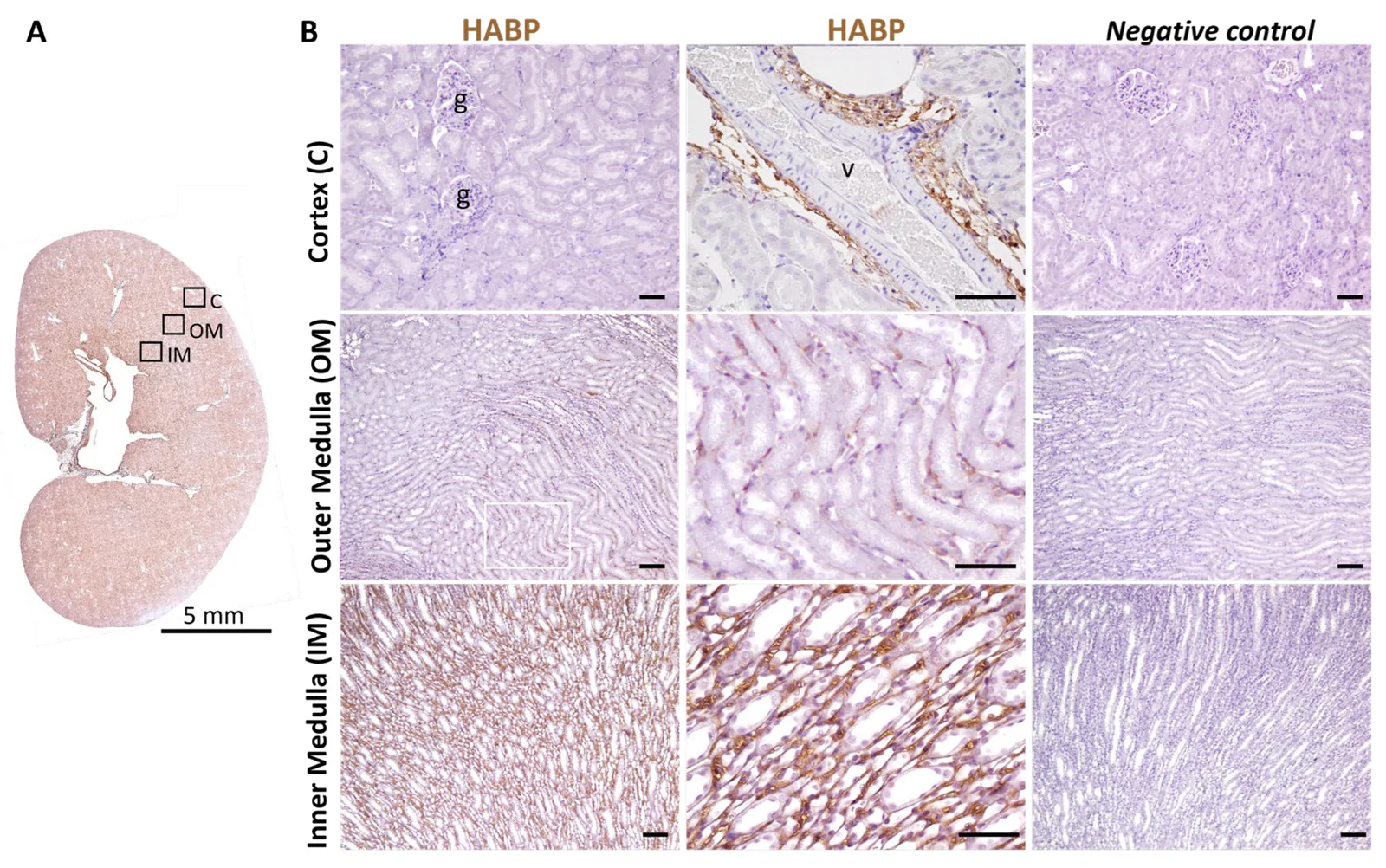
Hyaluronan (HA) localization in the healthy adult rat kidney. (**A**) Coronal kidney section with boxed regions indicating the cortex (C), outer medulla (OM), and inner medulla (IM). Higher-magnification images of each region are shown in (**B**). (**B**) Histochemical detection of HA using biotinylated hyaluronan-binding protein (HABP) and avidin–biotin horseradish peroxidase (HRP) detection. HA is largely absent from the cortex, with limited perivascular staining surrounding larger blood vessel and arterioles. HA staining progressively increases from the outer to the inner medulla, where it is localized predominantly within the interstitium between tubules. Negative control with HABP omitted. g—glomerulus, v—blood vessel. Scale bars, 50 μm. The white box indicates the region shown at higher magnification in the adjacent image.

Hyaluronan (HA) has low immunogenicity and cannot be detected directly by antibodies but can be visualized using a hyaluronan-binding protein (HABP) derived from bovine nasal cartilage. In healthy, adult rat kidneys, HA was largely absent from the renal cortex, with staining restricted to the perivascular stroma surrounding larger blood vessels and arterioles (Figure 1). No HA was detected in the peri-glomerular, peri-tubular, or cortical interstitial regions. In contrast, HA staining progressively increased from the outer medulla to the inner medulla and papilla, where it was localized predominantly within the interstitium between tubules and capillaries, surrounding the loops of Henle. This distribution is consistent with previous reports suggesting a role for medullary HA in urine concentration [36].

### 3.2. HA Demonstrates Increased Stromal Deposition Following IRI and Localizes in Areas of α-SMA+ Myofibroblast Expansion

**Figure 2:**
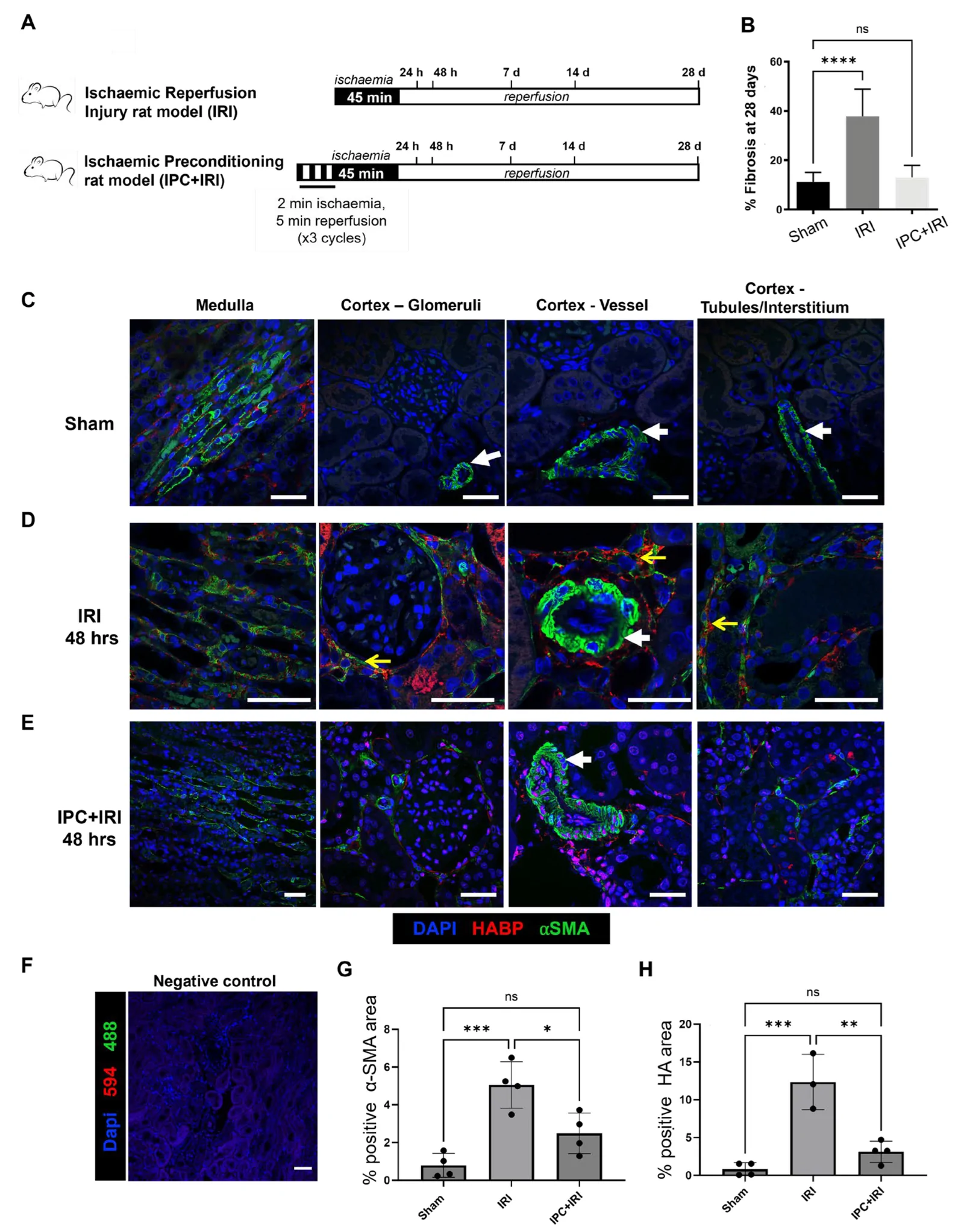
Increased HA deposition in the renal interstitial stroma following progressive damage correlates with increased myofibroblast positive regions. (**A**) Schematic of Ischaemia–Reperfusion Injury (IRI) model and Ischaemic Pre-Conditioning (IPC) model in rats. Adult male Lewis rats (*n* = 72) underwent a midline laparotomy and were divided into two groups; sham operation (*n* = 35), bilateral IRI (cross-clamping of both renal pedicles for 45 min) (*n* = 37) or IPC + IRI (3 cycles of ischaemic pre-conditioning prior to IRI: 2 min ischaemia and 5 min reperfusion) (*n* = 24). The groups were further subdivided depending on their post-operative observation time: 24 h (sham *n* = 8, IRI *n* = 8, IPC + IRI *n* = 8), 48 h (sham *n* = 11, IRI *n* = 13), 7 days (sham *n* = 4, IRI *n* = 4, IPC + IRI *n* = 4), 14 days (sham *n* = 4, IRI *n* = 4, IPC + IRI *n* = 4) and 28 days (sham *n* = 8, IRI *n* = 8, IPC + IRI *n* = 8). At the designated post-operative time, kidney tissue was recovered under terminal anesthesia. (**B**) IPC attenuates cortical interstitial fibrosis at 28 days post injury. (**C**) Kidney immunofluorescence sections in (**C**) normal (sham), (**D**) IRI and (**E**) IPC + IRI demonstrating HA (red) αSMA (green) and nuclear (blue) staining. White arrowheads denote αSMA (green) staining of vessel walls. Yellow arrows denote areas of combined αSMA (green) and HABP (red) staining in the renal interstitium. Scale bars 50 μm, Original Magnification ×400. (**F**) Representative image of a negative control in which the primary antibody and HABP was omitted, demonstrating minimal background fluorescence in the 488 and 596 nm channels. Scale bar 50 μm. (**G**) Quantification of the percentage area occupied by α-SMA-positive immunofluorescence in sham, IRI, and IPC + IRI kidneys at 48 h (*n* = 3–4 rats per group; 10 fields of view (FOVs) analyzed per kidney). (**H**) Quantification of the percentage area occupied by HABP-positive fluorescence in sham, IRI, and IPC + IRI kidneys at 48 h (*n* = 3–4 rats per group; 10–12 FOVs analyzed per kidney). Statistical significance is denoted as * *p* < 0.05; ** *p* < 0.01; *** *p* < 0.001 and **** *p* < 0.0001, ‘ns’ indicates statistical non-significance.

Animals underwent IRI through clamping of both renal pedicles for 45 min. The clamps were then removed, wounds closed, and animals recovered and maintained for 2, 7, 14 and 28 days post-operatively before kidney tissue was harvested. An experimental group of animals underwent ischaemic pre-conditioning (IPC) prior to IRI (schematic of model demonstrated in Figure 2A). IPC has been shown to attenuate the deleterious effects of IRI and thus represents a renal recovery model [28]. Our studies demonstrated that IRI led to both acute renal injury demonstrated by elevated serum creatinine levels and chronic interstitial fibrosis [29]. In contrast, we showed that ischaemic pre-conditioning (IPC) prior to IRI led to enhanced recovery from IRI acutely and from chronic renal interstitial fibrosis (Figure 2B) [28,29].

To determine the renal expression and localisation of HA, IF was performed on rat kidney sections following 48 h of IRI. Our previous in vitro data in fibroblasts indicated that the generation of pericellular HA matrices is critical for differentiation to and maintenance of pro-fibrotic α-SMA+ myofibroblast phenotypes [26]. Hence, co-staining for α-SMA was performed to determine the relationship between the localisation of HA and α-SMA+ myofibroblasts in vivo.

In healthy (sham) kidneys, HA (red) was observed surrounding the loops of Henle in the renal medulla. Little HA was seen in the glomerular, perivascular or tubular/interstitial areas of the renal cortex. Furthermore, there was no interstitial α-SMA+ (green) staining in healthy kidneys, indicating the absence of pro-fibrotic myofibroblasts. The α-SMA staining in healthy kidneys was limited to the walls of the renal arteries/arterioles, where it stained contractile α-SMA+ vascular smooth muscle cells (Figure 2C). Following IRI, HA deposition was increased in the renal cortex and was particularly evident in peri-glomerular, perivascular and peri-tubular interstitium. Staining for α-SMA+ myofibroblasts was also abundant in these HA-enriched areas (Figure 2D). This spatial association is consistent with our previous in vitro studies, which demonstrated a causal role for HA pericellular matrices in driving myofibroblast differentiation. Application of IPC prior to IRI resulted in a considerable change to HA and α-SMA localisation in the renal cortex, with only modest expression of both now present within the renal stroma (Figure 2E–H).

### 3.3. Kidney Stromal HA Demonstrates Distinctive Patterns of Expression and Localization During Development, in Health and After Injury

HA has an established role in embryonic development, with *Has2*^−/−^ mice demonstrating lethal cardiovascular anomalies [37]. To gain insights into the role of HA in kidney development, E15.5 mouse kidneys were stained for HA. At E15.5, the overall nephron segments are specified, but new nephrons continue to be generated from nephron progenitors [38]. Therefore, insights into the stromal environment that supports nephron regeneration were assessed at this stage to enable subsequent comparisons to be made with stroma in mature kidneys. The results demonstrated abundance of HA throughout the developing kidney with expression evident in the nephrogenic zone (nz), surrounding the ureteric bud (ub) branches, in the cortical stroma (cs) grouped around developing tubules, and in the medullary stroma (ms) (Supplementary Figure S1A). To determine the HAS isoenzyme responsible for HA generation in the developing kidney, HAS1 and HAS2 immunohistochemistry was performed. HAS1 was detected in a subset of tubular and stromal cells (Supplementary Figure S1B), but HAS2 was not found (Supplementary Figure S1C). IF analysis demonstrated co-localisation of HA (green) with HAS1 (red) in the stroma (Supplementary Figure S1A), suggesting HAS1 plays a prominent role in stromal HA generation in kidney development.

**Figure 3:**
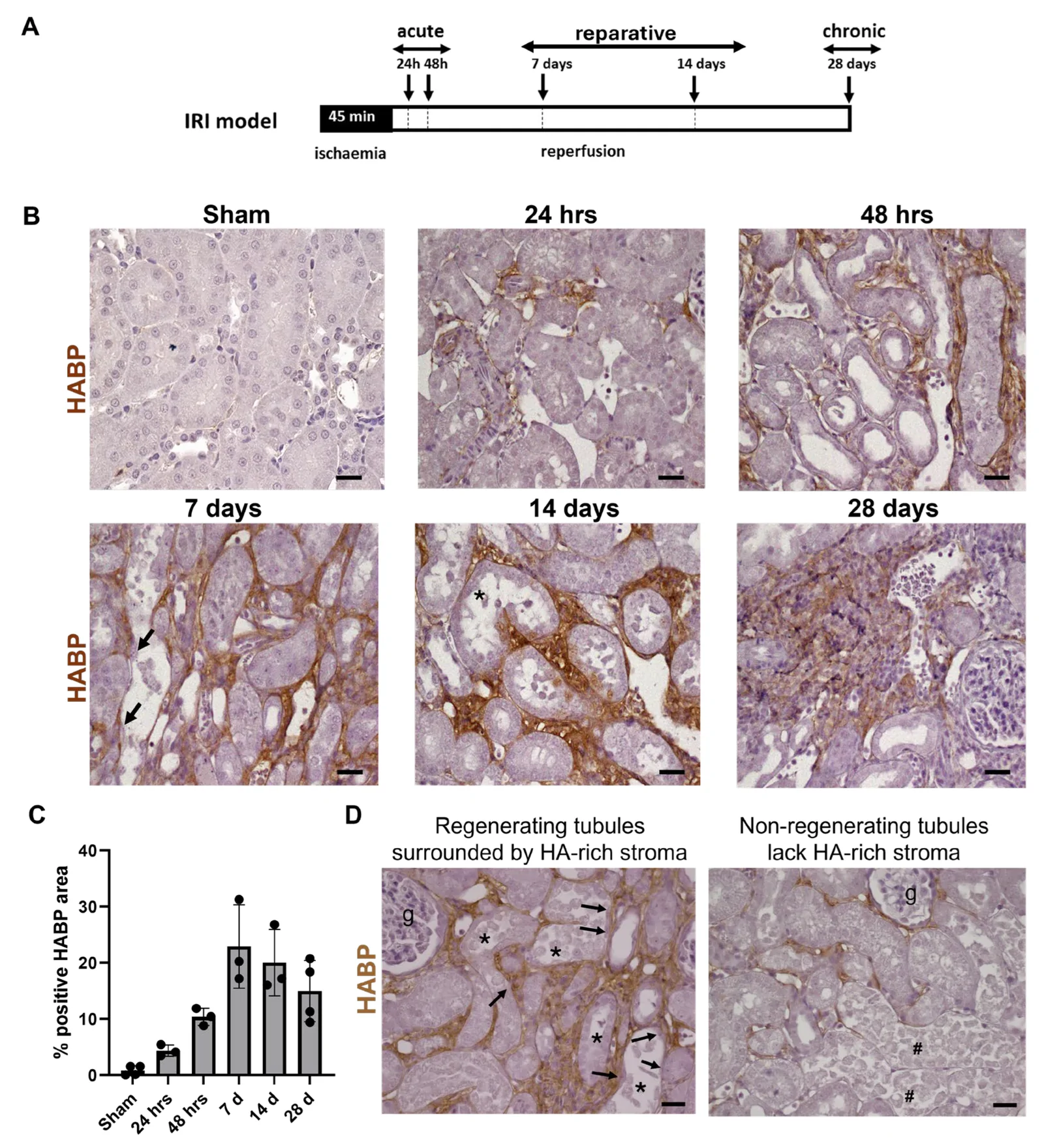
(**A**) Schematic of the rat renal ischaemia–reperfusion injury (IRI) model showing the tissue-collection time points for histological analysis. (**B**) Representative images of hyaluronan-binding protein (HABP) staining in the renal cortex at different time points following IRI. (**C**) Quantification of the HABP-positive area (*n* = 3–4 rats per group; 10 fields of view (FOVs) analyzed per kidney). (**D**) Representative images of HABP staining in kidneys 48 h after IRI showing discrete HA-rich stromal niches. Abundant stromal HA is localized adjacent to regenerating tubules (*), characterized by flattened epithelial cells (black arrows), whereas tubules undergoing extensive necrosis with no evidence of regeneration (#) lack surrounding HA-rich stroma. Scale bars, 20 μm. The label “g” identifies a glomerulus.

Staining for HABP was undertaken in healthy kidneys and in kidneys 24 h to 28 days post-IRI (Figure 3). Our recent studies indicate that in this model there is prominent acute renal injury at 24 and 48 h, which begins to subside at 7 days. At 7 days and 14 days there was also evidence that tubules were undergoing repair, whilst at 28 days post-IRI, florid fibrosis and tubulointerstitial damage were observed [28,29]. In view of this, we designated the 24–48 h period post-IRI as the acute phase, 7–14 days post-IRI as the regenerative phase, and the 28-day period as the chronic fibrosis phase.

HA was absent from the renal cortex in health but was rapidly expressed in the renal stroma following ischaemia, with staining evident at 24 h post-injury localizing to perivascular interstitium of larger vessels as well as smaller arterioles in the cortex where HA is not normally present in homeostasis (Figure 3). Stromal HA was increased from 24 h up to 7 days and was prominent in areas surrounding repairing tubules (black arrows) and in capillary dense regions (Figure 3B,D). This bore a striking resemblance to stromal HA distribution in the developing kidney (Supplementary Figure S1), where stromal HA also surrounded areas of tubular development and new capillary formation. At 14 days post-IRI, stromal HA was becoming less prominent and by day 28, HA was present in stromal matrices only in areas of unresolved inflammation and fibrotic scarring.

### 3.4. Distinct HA Synthase Isoenzymes Show Differential Patterns of Expression, and Kidneys Expressing Only Has2 Are Predisposed to Developing Fibrosis

**Figure 4:**
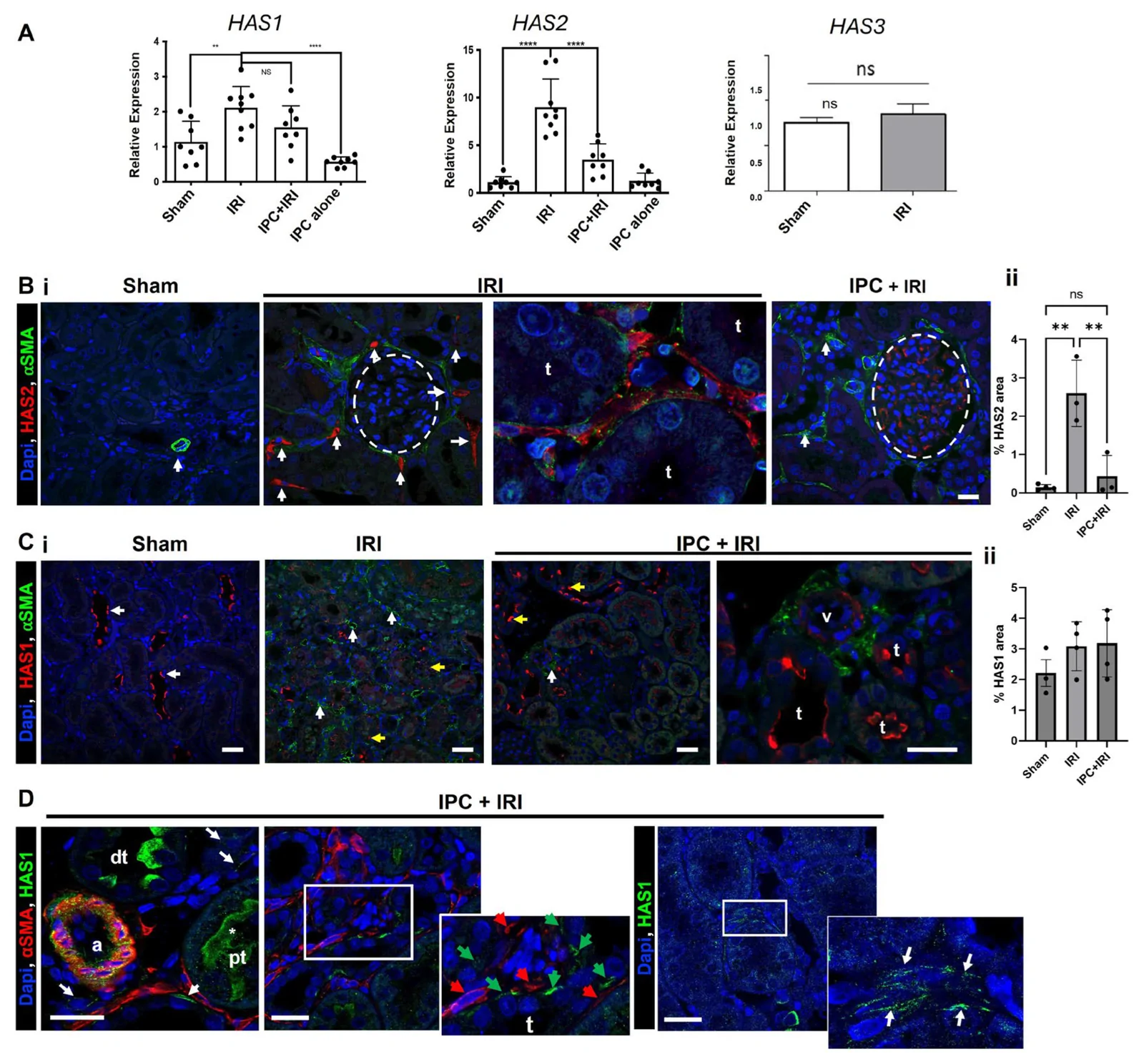
HAS1 and HAS2 demonstrate differential expression patterns in normal kidneys and kidneys that only express the HAS2 isoenzyme demonstrate altered patterns of eCadherin, α-SMA and HA. (**A**) Kidneys from normal (Sham), IRI alone and IPC + IRI models were assessed using RT-qPCR of whole kidney to assess expression of *Has1*, *Has2*, and *Has3* mRNA. (**B**) (**i**) Immunofluorescence detection of HAS2 (red) and α-SMA (green) in normal (sham), IRI-only and IRI pre-treated with IPC rat kidneys. Nuclei are stained with DAPI (blue). White arrow in sham image denotes staining of small blood vessels with α-SMA. White arrows in IRI images denote HAS2 staining of stromal cells in the interstitium. Dashed white circles highlight the glomeruli. (**ii**) Quantification of the percentage area occupied by HAS2-positive fluorescence in sham, IRI, and IPC + IRI kidneys at 48 h (*n* = 3–4 rats per group; 10–12 FOVs analyzed per kidney). (**C**) (**i**) Immunofluorescence detection of HAS1 (red) and α-SMA (green) in normal (sham), IRI-only and IRI pre-treated with IPC rat kidneys. Nuclei are stained with DAPI (blue). White arrows denote increased α-SMA (green) staining in areas of damage/fibrosis. Yellow arrows demonstrate HAS1 (red) expression at the apical aspect of a proportion of tubular epithelial cells. (**ii**) Quantification of the percentage area occupied by HAS1-positive fluorescence in sham, IRI, and IPC + IRI kidneys at 48 h (*n* = 3–4 rats per group; 10–12 FOVs analyzed per kidney). (**D**) Higher-magnification images of rat kidneys treated with IPC prior to IRI stained for HAS1 (green), α-SMA (red) and DAPI (nuclei, blue). Increased HAS1 positive staining can be identified in the arterioles (a) at the apical border of distal tubular epithelial cells (dt) and in interstitial stromal cells (white arrows). In the last panel, α-SMA positive stromal cells (red) and HAS1 positive stromal cells (green) are identified by red and green arrowheads respectively to distinguish them. Scale bars, 20 μm. qPCR data are presented as dot plots representative of 3 separate experiments. ** *p* < 0.01; **** *p* < 0.0001, ‘ns’ indicates statistical non-significance. Scale bars 50 μm, Original Magnification ×400. The label “t” identifies a tubule, “v” identifies a vessel, “pt” identifies a proximal tubule and “dt” identifies a distal tubule.

Synthesis of HA is dependent on three membrane-bound HA synthase enzymes: HAS1, HAS2 and HAS3. The role of these distinct isoenzymes in the generation of stromal HA matrices in mature kidneys in health, following ischaemic injury and during recovery from ischaemic injury, was investigated using our models of IRI +/− IPC. mRNA expression of the *Has* isoenzyme mRNA was assessed using RT-qPCR of the whole kidney. The data demonstrated that there was a 1.5–2-fold induction of *Has1* expression following IRI. In IPC + IRI animals, renal expression of *Has1* remained elevated. In contrast, *Has2* mRNA expression increased markedly compared to *Has1* expression following IRI, with a 9–10-fold induction in expression. In IPC + IRI, *Has2* mRNA expression was attenuated, suggesting that HAS2 was the principal profibrotic isoenzyme (Figure 4A). *Has3* mRNA expression was unchanged in response to injury (Figure 4A) and across all cohorts.

IF detection of HAS1 and HAS2 isoenzymes was subsequently undertaken. Initially, dual-staining compared HAS2 (red) and α-SMA+ (green) myofibroblast localisation (Figure 4B). As previously demonstrated, α-SMA+ staining was observed only in the vessel walls in sham animals. However, following IRI, α-SMA+ staining was identified throughout the interstitium. In IPC + IRI animals, interstitial α-SMA+ staining was attenuated. In sham animals, HAS2 was not identified in the renal cortex, whilst there was a noticeable increase in interstitial HAS2 in IRI animals. The HAS2+ cells localized to regions enriched with α-SMA+ cells. In accordance with Has2 gene expression, HAS2 protein expression was also attenuated in the interstitial stroma in IPC + IRI.

HAS1 (red) localisation in the kidney was explored next (Figure 4C). In sham kidneys, HAS1 was only present at the apical border of tubular epithelial cells in a subset of tubules comprising approximately 30–40 percent of the renal cortex. Following IRI, HAS1 immunoreactivity decreased, and the distinct localisation seen in the sham animals was disrupted. As expected, extensive interstitial α-SMA (green) was also present following IRI; however, unlike with HAS2, α-SMA and HAS1 were not identified in the same areas, with no HAS1 demonstrated in the interstitial space. In animals pre-treated with IPC, HAS1 demonstrated apical tubular cell expression in a subset of tubules, and this was marginally enhanced compared to sham/healthy kidneys. Moreover, HAS1 was also present in other non-tubular cell types including expression in vascular smooth muscle cells and in a subpopulation of interstitial stromal cells comparable to our observations during kidney development. Of note, interstitial HAS1+ cell staining in IPC + IRI-treated animals was distinct from α-SMA+ interstitial cell staining (red versus green arrows) (Figure 4D).

**Figure 5:**
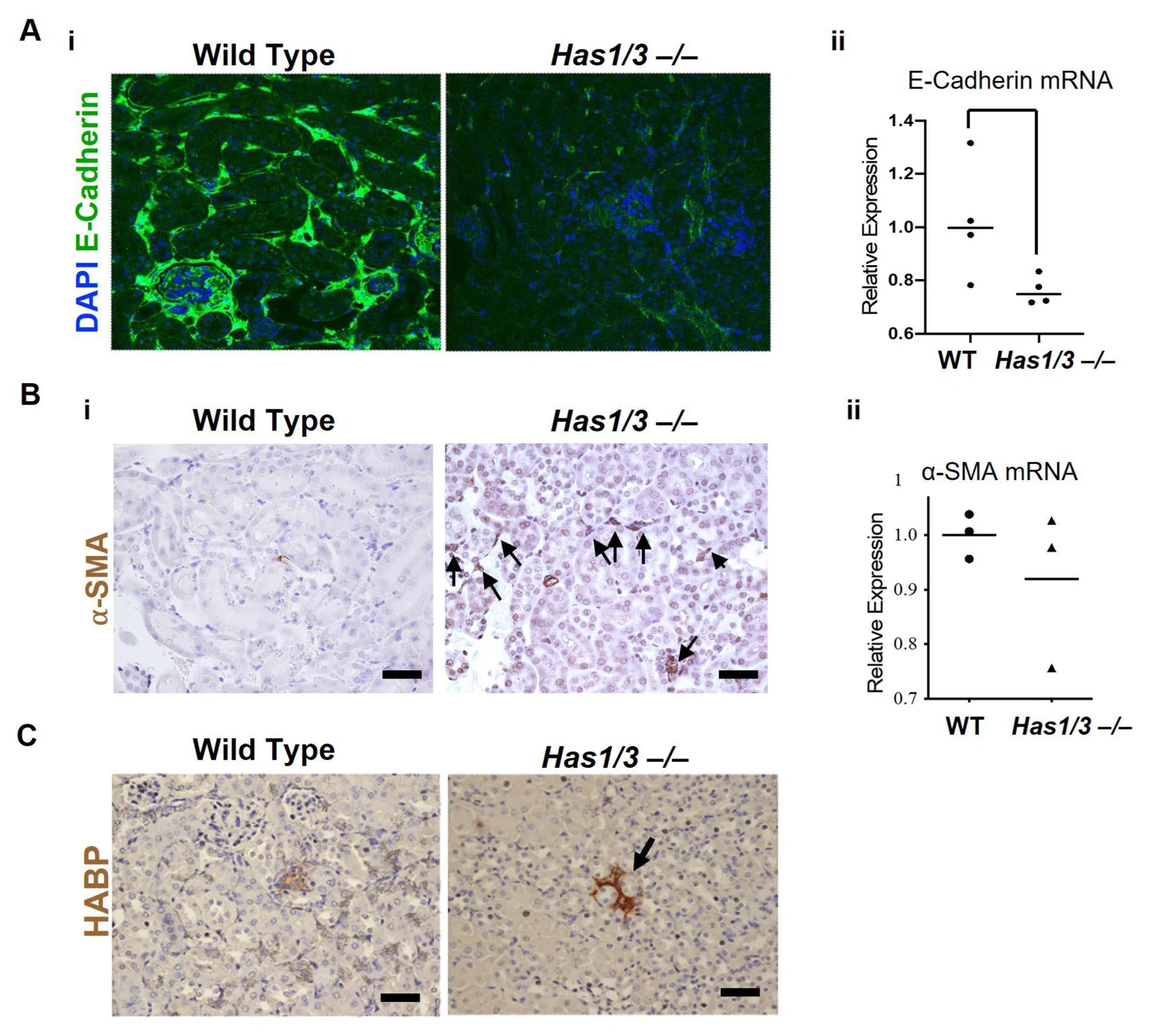
Kidneys expressing only HAS2 are predisposed to developing fibrosis. Kidneys from 12-week-old C57BL6 Wild Type (*n* = 3) and *Has1/3* double-knockout (*n* = 3) mice were harvested and shipped by Professor A. Plaas (Rush University, Chicago, IL, USA). Kidney specimens in RNAlater were homogenized and RNA was extracted from whole tissue for RT-qPCR analysis. Kidneys embedded in paraffin were sectioned, deparaffinized and histological sections prepared for immunofluorescence/immunohistology staining. (**A**) (**i**) Representative immunofluorescence images showing E-cadherin expression in wild-type and *Has1/3* double-knockout kidneys; (**ii**) relative E-cadherin mRNA expression assessed by qPCR. (**B**) (**i**) Representative immunohistochemical images showing α-SMA expression in wild-type and *Has1/3* double-knockout kidneys; black arrows indicate areas of increased α-SMA staining; (**ii**) relative α-SMA mRNA expression assessed by qPCR. (**C**) Representative immunohistochemical images showing HABP staining in wild-type and *Has1/3* double-knockout kidneys; the black arrow denotes a small pocket of interstitial HA staining. qPCR data are presented as dot plots representative of three separate experiments. Scale bars, 50 μm. Original magnification, ×400.

Next, mouse kidneys with a functional *Has1*^−/−^*Has3*^−/−^ double-knockout (*Has1/3*^−/−^) were investigated both histologically and through RT-qPCR for markers of fibrosis. *Has2* knockout mice demonstrate embryonic lethality and could not be analyzed. Naïve *Has1/3*^−/−^ mice had normal renal histology, but reduced E-cadherin staining and mRNA expression was observed (Figure 5A(i,ii)). These animals also demonstrated areas of interstitial α-SMA+ myofibroblast staining (Figure 5B(i)), although whole-kidney α-SMA mRNA detection was not significantly different to wild-type (Figure 5B(ii)). Moreover, *Has1/3*^−/−^ mice demonstrated small pockets of stromal HA staining in the renal cortex, which were absent in wild-type mice (Figure 5C), suggesting aberrant HAS2-driven HA synthesis in the renal cortex.

### 3.5. HAS1+ and HAS2+ Fibroblasts Display Distinct Pericellular Matrices

**Figure 6:**
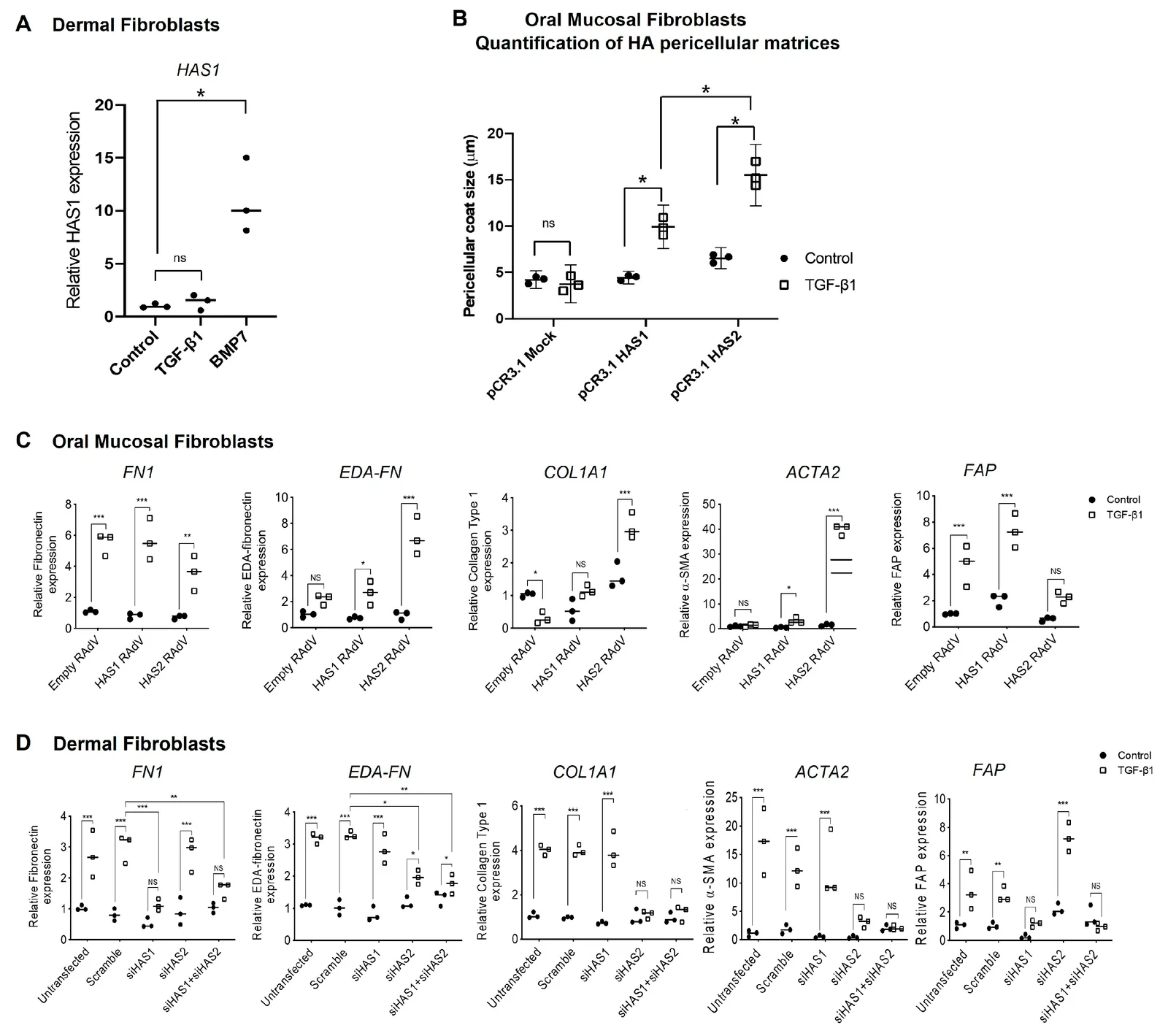
HAS1 and HAS2 isoenzymes are associated with distinct pericellular matrices and phenotypic characteristics. (**A**) Dermal fibroblasts are stimulated with either serum-free medium (control), 10 ng/mL TGF-β1 or 200 ng/mL of BMP7 for 72 h. RT-qPCR is used to determine mRNA expression of HAS1. (**B**) Empty recombinant adenoviral vector (RAdV) and *HAS1* or *HAS2* RAdVs were used to infect oral mucosal fibroblasts in the absence (●) or presence (□) of 10 ng/mL TGF-β1 for 72 h. (**B**) Pericellular matrices HA were investigated using the red cell exclusion assay and observed using light microscopy and pericellular HA coat sizes were calculated by image analysis of OMFs following the HAS expression vector treatments described above. (**C**) Empty recombinant adenoviral vector (RAdV) and *HAS1* or *HAS2* RAdVs were used to infect oral mucosal fibroblasts in the absence or presence of 10 ng/mL TGF-β1 for 72 h. RT-qPCR was used to assess mRNA expression of Fibronectin (*FN1*), EDA-splice variant fibronectin (EDA-FN) and collagen-1 (*COL1A1*), α-SMA and Fibroblast Activated Protein (*FAP*). (**D**) Dermal fibroblasts were transfected with scrambled siRNA or siRNA targeting *HAS1*, *HAS2* or both and incubated in the absence or presence of 10 ng/mL TGF-β1 for 72 h. RT-qPCR was used to assess mRNA expression of *FN1*, EDA-FN, *COL1A1*, α-SMA and *FAP*. Data are expressed as dot plots and are representative of three independent biological experiments using fibroblasts from three separate donor-matched oral mucosal and dermal fibroblasts. * *p* < 0.05; ** *p* < 0.01; *** *p* < 0.001; ‘ns’ indicates statistical non-significance.

Compared to other adult human tissues, the oral mucosa undergoes scarless healing [33]. Indeed, fibroblasts from the oral mucosa demonstrate intrinsic biological properties that explain their propensity for scar-free healing. Specifically, oral mucosal fibroblasts are resistant to TGF-β1-driven myofibroblast differentiation and proliferation [26,34]. To better understand the cellular biology involved in scarring/fibrosis versus scar-free healing, we used donor-matched oral mucosal and dermal fibroblasts as models of non-scarring and scarring fibroblast phenotypes respectively. In initial experiments, stimulation of dermal (scar-forming) fibroblast phenotypes with TGF-β1 led to limited/no upregulation of *HAS1* mRNA. In contrast, in the presence of anti-fibrotic growth factor bone morphogenic protein-7 (BMP7), marked *HAS1* upregulation was observed (Figure 6A), indicating a role for HAS1 as a downstream mediator of BMP7 reno-protection.

We then focused on identifying specific cellular functions for HAS1 versus HAS2. We have shown that TGF-β1-driven responses in oral mucosal and dermal fibroblasts are causally linked to the cells’ ability to generate pericellular HA matrices, essential for differentiation to and maintenance of a pro-fibrotic α-SMA+ myofibroblast phenotype [24,26,32]. Our data has fully characterized the HA profile of these distinct fibroblast phenotypes, showing that dermal fibroblasts express both HAS1 and HAS2 isoenzymes, whereas oral fibroblasts exhibit only low-level HAS2 expression [26]. In this study, we used adenoviral vectors to force expression of HAS1 or HAS2 in oral mucosal fibroblasts, thereby enabling evaluation of the effects of these distinct isoenzymes on TGF-β1 responses in fibroblasts. We also used siRNA to abrogate *HAS1* and/or *HAS2* in dermal fibroblasts. Initial experiments were undertaken to confirm HAS1 and HAS2 over-expression and/or knockdown (Supplementary Figure S2).

The red-cell exclusion assay was used to assess the effects of forced *HAS1 versus HAS2* expression on oral fibroblast HA pericellular matrix formation. HAS1+ fibroblasts generated modest HA pericellular matrices, contrasting with the larger exclusion zones surrounding HAS2+ fibroblasts (Figure 6B and Supplementary Figure S3). RT-qPCR was used to analyze the expression of ECM components in HAS1+ versus HAS2+ cells. HAS2+ fibroblasts showed significantly upregulated EDA-fibronectin (*EDA-FN*) and Collagen-AI (*COL1A1*) mRNA expression. In contrast, in HAS1+ fibroblasts detection of non-EDA fibronectin increased and *EDA-FN* and *COL1A1* decreased (Figure 6C). Conversely, siRNA-mediated knockdown of *HAS1* versus *HAS2* undertaken in dermal fibroblasts demonstrated that HAS2 knockdown led to marked attenuation of *EDA-FN* and *COL1A1*, whereas HAS1 knockdown led downregulation of *FN1* expression (Figure 6D).

### 3.6. HAS1+ and HAS2+ Fibroblasts Display Distinct TGF-β1-Driven Phenotypes

Gene expression of two stromal markers was compared in HAS1+ versus HAS2+ fibroblasts. Detection of α-SMA (*ACTA2*) was quantified to determine differentiation to the classical pro-fibrotic myofibroblast phenotype. In addition, mRNA expression of the cell-surface protease Fibroblast Activated Protein (*FAP*) was analyzed. FAP has been identified as a stromal marker that provides protection from fibrotic damage in lung fibrosis, through its ability to break down extracellular matrix [39].

In our cells, forced HAS1 expression in oral fibroblasts led to a small increase in *ACTA2* expression, but a four-fold increase was observed following HAS2 overexpression (Figure 6C). In dermal fibroblasts, HAS1 knockdown had only a marginal effect on *ACTA2* expression, whereas a marked attenuation of *ACTA2* expression followed *HAS2* knockdown (Figure 6D). Conversely, forced HAS1 expression enhanced *FAP* levels in oral fibroblasts compared to forced HAS2 expression (Figure 6C). Moreover, *HAS1* knockdown had a more pronounced effect on *FAP* mRNA expression in dermal fibroblasts compared to *HAS2* knockdown (Figure 6D).

### 3.7. HAS1+ Fibroblasts Are More Pro-Migratory in Comparison to HAS2+ Fibroblasts That Display a More Pro-Contractile Phenotype

**Figure 7:**
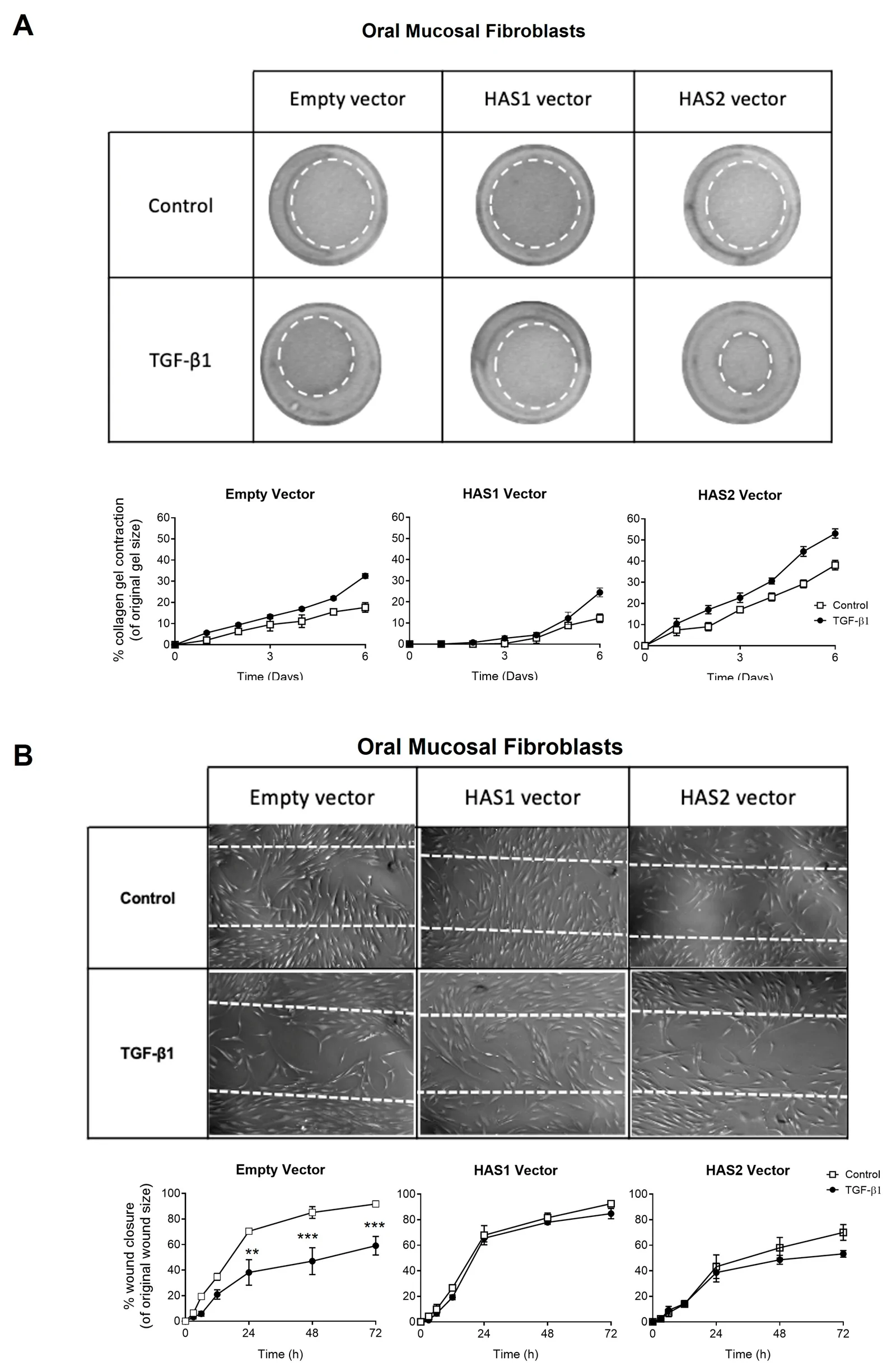
HAS1 and HAS2 isoenzymes have distinct effects on fibroblast contraction and migration. Empty recombinant adenoviral vector (RAdV) and HAS1 or HAS2 RAdVs were used to infect oral mucosal fibroblasts. (**A**) Cells were seeded into collagen gels and left to polymerize before incubation in the absence or presence of 10 ng/mL TGF-β1. Gels were imaged over the annotated time points and measured for analysis of rate of contraction. Dotted circles indicate the measured gel areas at this time point and percentage collagen gel contraction of original gel size is quantified at each time and annotated as a graph. (**B**) Cells were growth-arrested and scratched using a 20 μL pipette tip. After a phosphate-buffered saline wash, the cells were incubated in the absence or presence of 10 ng/mL TGF-β1. Scratch assays were imaged at the indicated time points, and the area of closure was measured to assess the rate of migration. Dotted lines represent the original scratch edges and percentage wound closure compared to original wound size is measured to quantify rate of wound closure and annotated as a graph. Data are representative of three independent biological experiments. ** *p* < 0.01; *** *p* < 0.001.

A well-established characteristic of the pro-fibrotic α-SMA+ myofibroblast phenotype is its pro-contractile properties. Hence, the role of HAS1 and HAS2 iso-enzymes on TGF-β1-driven contractility was investigated. Oral mucosal fibroblasts over-expressing HAS1 had attenuated contractility compared to oral fibroblasts expressing HAS2 (Figure 7A). To quantify this, the diameter of collagen-gels was measured every 24 h. The results indicated a statistically significant increase in collagen-gel contraction following HAS2 over-expression.

FAP facilitates stromal cell migration through tissue matrix degradation [39]. Since our data indicated differences in *FAP* expression (Figure 6C,D), we next analyzed the role of HAS isoenzymes in fibroblast migration. These data showed that TGF-β1 had an anti-migratory effect in un-transfected oral fibroblasts: forced HAS1 expression attenuated the anti-migratory TGF-β1 effects, whereas HAS2 over-expression had limited impact on this (Figure 7B).

### 3.8. Alignment of HAS Genomic DNA Sequences Reveals a Conserved Noncoding Region (CNR) of Upstream Sequence Shared by HAS1 and HAS2

Analysis of 3kb of genomic DNA sequence upstream of the transcription start sites of *HAS1*, *HAS2* and *HAS3* revealed a single CNR with >70% nucleotide sequence conservation shared between *HAS1* and *HAS2* and spanned 165 bp (Supplementary Figure S4A,B).

To investigate the evolutionary history of this CNR, we reconstructed branch lengths from a midpoint rooted tree using maximum likelihood as implemented by TreeFinder and 1000 bootstrap replicates [40]. The HAS2 and HAS3 sequences were more closely related to each other than either was to *HAS1*, and the CNR was lost in the *HAS3* lineage (Supplementary Figure S4C).

## 4. Discussion

HA is a non-sulphated glycosaminoglycan that forms a key structural component of the ECM. HA is ubiquitously expressed and interacts directly with cells regulating processes including cell–cell adhesion and crosstalk, migration, proliferation, and differentiation [41,42,43,44,45]. HA is therefore implicated in influencing numerous biological processes and has a vital role during mammalian organogenesis and development. Therefore, it is no surprise that dysregulated HA synthesis, turnover and binding interactions contribute to numerous disease states, including atherosclerosis, chronic inflammation, cancer and fibrosis [46,47,48,49].

In this study, we used the developing kidney as a regenerative reference state to determine whether stromal HA patterns associated with nephrogenesis are recapitulated during repair following adult kidney injury. During embryonic kidney development, there is abundant HA in the stroma of the renal cortex. HA was absent from the mature healthy kidney cortex, but was rapidly re-expressed in the interstitial stroma following ischaemic injury. Furthermore, in mature kidneys protected from ischaemic damage, there was attenuated interstitial HA in the stroma. These collective findings suggest that renal HA may have dual and conflicting roles: stromal HA potentially plays an important role in nephrogenesis and in tubular regeneration following injury; however, persistence of stromal HA following injury promotes progressive fibrosis leading to nephron destruction rather than regeneration. Thus, understanding how developmental HA programmes are recapitulated or diverge in development versus adult injury may provide clues towards HA modification to promote regeneration rather than fibrosis.

HA is synthesized by the iso-enzymes HAS1, HAS2 and HAS3. The principal cell- surface HA receptor is CD44, but HA can also bind to a number of HA-binding proteins called haladherins. The biological functions of HA are determined by its interactions with these hyaladherins, as well as its molecular weight and manner of synthesis and assembly. Thus, the biology of HA is complex, and it can have diverse and sometimes opposing functions depending on these factors.

Deletion of *Has2* in mouse embryos results in growth retardation evident by embryonic day 9 and the mice die at mid-gestation (before metanephric kidney development and ureteric bud outgrowth) [37]. By contrast, mice lacking one or both *Has1* and *Has3* genes are viable and exhibit no obvious phenotype [50,51].

Our previous cell studies outlined the pathway via which HA regulates differentiation to an α-SMA+ myofibroblast phenotype, outlining the importance of a HAS2-generated cell-surface “HA coat” tethered to CD44 complexed with CD147 within the cell membrane [52]. This promoted MAPK/ERK signalling, which alongside SMAD signalling was critical for differentiation to and maintenance of the pro-fibrotic α-SMA+ myofibroblast phenotype [23,27,32,53]. Whilst the current findings are consistent with our previous mechanistic studies, direct investigation of CD44-, ERK/MAPK- and TGF-β-dependent signalling in vivo was beyond the scope of this study and represents an important area for future investigation. Our studies also showed that cytoplasmic internalization and degradation of the HA coat can both prevent and reverse α-SMA+ differentiation, indicating that continued presence of a HA matrix can promote pro-fibrotic effects at a cellular level [25,54].

This study builds on our previous cell studies, demonstrating that increased HAS2 and interstitial HA in the renal cortex in vivo correlates with increased interstitial α-SMA+ myofibroblasts and promotes pro-fibrotic outcomes. We also confirm that fibroblasts with augmented HAS2 expression are associated with a contractile phenotype and display enhanced expression of pro-fibrotic markers including EDA-FN and *COL1A1*, whilst having attenuated expression of protective proteins such as *FAP*. Interestingly, genetically modified mice only capable of HAS2-driven HA generation appeared to be primed towards developing renal injury with increased interstitial pockets of α-SMA and HA, even without renal injury. These findings are in keeping with studies looking at the fibrotic effects of HAS2 in models of liver, skin and lung fibrosis [55,56,57,58]. A limitation of this study is the relatively small sample size for the Has1/3 double-knockout mice, and these findings warrant validation in larger genetically modified cohorts.

In contrast to HAS2, the literature on HAS1 and its role in HA generation, particularly in the kidneys, is limited. HAS1 has received the least attention with its biological effects on cellular behaviour and association with disease states remaining largely unknown, and why vertebrates have three distinct HAS isoforms that are conserved through evolution remains an enigma.

Early studies suggested that HAS1 has the lowest activity of all the HAS enzymes and its role in biology may be limited to providing compensatory HA synthesis for HAS2 and HAS3, which have been depicted as the most active and biologically crucial HAS enzymes [59]. However, our report indicates that HAS1 has distinct biological functions from HAS2 in the kidney and in stromal cells. HAS1 is widely expressed and present during embryonic kidney development, particularly where HA is abundant in the stroma around developing tubules and areas of new capillary formation. In healthy mature kidneys, HAS1 has limited expression in the apical aspect of distal tubular epithelial cells, whilst in IRI-damaged kidneys, its expression is attenuated, disordered, and notably absent from areas of increased HA expression where HAS2 predominates. This indicates that HAS1 is not responsible for stromal HA synthesis during renal injury.

However, in our protective renal model, HAS1 is enhanced in the vasculature, in distal tubular epithelial cells and notably in a population of interstitial cells that is distinct from α-SMA+ myofibroblasts. In cell studies, *HAS1* expression was markedly enhanced following stimulation with the reno-protective cytokine BMP7. HAS1+ fibroblasts also had attenuated expression of pro-fibrotic markers *ACTA2*, *EDA-FN* and *COL1A1*, and were less contractile, but demonstrated increased *FAP* expression and were more pro-migratory.

FAP is known to mediate collagen clearance in models of lung disease, leading to resolution of fibrous scarring [39]. Thus, the increased expression of HAS1 in IPC, the BMP7-mediated upregulation of *HAS1* expression and the augmented FAP expression in HAS1+ fibroblasts (alongside attenuated expression of pro-fibrotic markers) collectively suggest that HAS1 is associated with a protective stromal phenotype in the kidney. Single-cell sequencing studies from both lung and kidney tissues supports the existence of distinct stromal fibroblast populations during fibrosis. In lung fibrosis, a HAS1+ stromal population is distinct from the α-SMA+/HAS2+ myofibroblast cluster [16], whilst recent kidney single-cell studies have similarly identified transcriptionally distinct activated fibroblast populations [60]. Future studies using cell-type-specific gain and loss-of-function approaches, or pharmacological modulation of individual HAS isoforms, will be important to define their causal roles in renal injury, repair and fibrosis in vivo.

Fibroblasts can specialize in different tissues and have distinct phenotypes depending on their site of isolation. Advances in lineage tracing and single-cell transcriptional profiling techniques have also revealed impressive diversity amongst fibroblasts in a range of organ systems including in the kidney [17]. In this report, not only do we demonstrate fibroblast subtypes as a useful tool to study stromal cell responses, but also demonstrate that modulation of HA, and in particular HAS isoenzymes, as an important regulator of fibroblast phenotypic heterogeneity highlighting HA biology as an important area for further study in the growing field of stromal cell heterogeneity.

## 5. Conclusions

In conclusion, this study demonstrates that HA, while largely absent in the healthy kidney, accumulates within the renal stroma during development, injury, and fibrotic progression, with dynamic changes in localisation and HAS expression across these states. We identify distinct roles for HAS isoenzymes, showing that HAS1 and HAS2 may be associated with different fibroblast phenotypes in vitro and occupy discrete stromal compartments and cell subpopulations in vivo. These findings provide new insight into HA-mediated regulation of stromal fibroblast heterogeneity, with potential relevance to fibrotic mechanisms in other organs. Importantly, we identify that HAS1+ stromal populations may be associated with renal recovery and protection from progressive fibrosis, highlighting HAS1-dependent pathways as potential therapeutic targets for promoting tissue repair and limiting chronic kidney disease progression.

## Data Availability

All data supporting this study is provided in full in the Section 3 or Supplementary Information accompanying this paper.

## Supplementary Materials

The following supporting information can be downloaded at: https://www.mdpi.com/article/10.3390/biom16101442/s1, Figure S1: (A) Immunofluorescence detection of HA binding protein (HABP, green) and HAS1 (red) in mouse embryonic day (E) 15.5 kidneys demonstrating abundance of HA and HAS1 in the stroma of the nephrogenic zone (nz) surrounding ureteric bud (ub) branches and induced nephrons in the cortical stroma (cs) grouping around sets of tubules, and in the stromal interstitium of the medulla. Boxed regions are depicted at higher magnification (lower panel) Scale bars 50 μm. Original Magnification x400. (B,C) Immunohistochemical detection of (B) HAS1 and (C) HAS2 demonstrating presence of HAS1 and absence of HAS2 in developing mouse kidneys at E15.5. Figure S2: Confirmation of *HAS* isoenzyme over-expression and knockdown. (A,B) Empty recombinant adenoviral vector (RAdV) and *HAS1* or *HAS2* RAdVs were used to infect oral mucosal fibroblasts in the absence (●) or presence (□) of 10 ng/ml TGF-b1 for 72 h. RT-qPCR was used to assess mRNA expression of *HAS1* and *HAS2* to confirm over-expression. Dermal fibroblasts were transfected with scrambled siRNA or siRNA targeting *HAS1*, *HAS2* or both and incubated in the absence (●) or presence (□) of 10ng/ml TGF-b1 for 72 h. RT-qPCR was used to assess mRNA expression of *HAS1 and HAS2*. Data are presented as dot plots representative of 3 separate experiments. ** *p* < 0.01; *** *p* < 0.001. (C,D) Dermal fibroblasts were transfected with scrambled siRNA or siRNA targeting *HAS1*, *HAS2* or both and incubated in the absence or presence of 10 ng/mL TGF-b1 for 72 h. RT-qPCR was used to assess mRNA expression of *HAS1* and *HAS2*. Figure S3: Pericellular matrices HA were investigated using the red cell exclusion assay and observed using light microscopy. Higher magnification images of boxed regions are show below; dashed line indicates the widest part of cell analysed, and solid line depicts the measurement taken for pericellular coat quantification. Figure S4: Human *HAS* gene promoter sequence alignment and phylogenetic HAS analysis. (A) VISTA output for a comparison of 3 kb upstream regions from *HAS1* (transcript *HAS1-001*; Ensembl genomic coordinates Chromosome 19: 52,216,365–52,227,247 reverse strand) and *HAS2* (transcript *HAS2-001*; Chromosome 8: 122,624,356- 122,653,630 reverse strand). Sequences were extracted from Ensembl release 70, which uses the GRCh37.p10 assembly. The red-shaded region represents a sequence >70% conserved between *HAS1* and *HAS2* and is shown with nucleotide positions relative to the *HAS1* transcription start site at 3001. (B) LAGAN alignment of the conserved region identified by VISTA, visualised using JALVIEW. (C) Phylogenetic analysis of human *HAS* gene cDNA sequences extracted from the Ensembl database. Sequences analysed were *HAS1-001* (ENST00000540069.2; labelled here as *HAS1*), *HAS2-001* (ENST00000303924.4; *HAS2*) and *HAS3-001* (ENST00000306560.1; *HAS3*). Numbers on the branches are branch lengths (sequence differences) from the midpoint of the tree, which is marked with an arrow. The presence or absence of the CNR is indicated by “+CNR” for presence and “-CNR” for absence. Inferred presence of the CNR at branch points is indicated in italics.

## Abbreviations

The following abbreviations are used in this manuscript:

α-SMA	Alpha Smooth Muscle Actin
CD44	Cluster of Differentiation 44
CKD	Chronic Kidney Disease
HA	Hyaluronan
HABP	Hyaluronan Binding Proteins
HAS	Hyaluronan Synthase
HYAL	Hyaluronidase
ORF	Open Reading Frame
TGF-β1	Transforming Growth Factor-β1
IRI	Ischaemia–reperfusion injury
IPC	Ischaemic pre-conditioning
