# Supplementary material for: Hyaluronan Synthase Isoforms Direct Distinct Patterns of Fibroblast Activation Linked to Fibrosis Progression Versus Resolution Following Renal Ischaemia
Source: Biomolecules. 2026 Oct 3;16(10):1442. doi: 10.3390/biom16101442 (PMC13702631; doi:10.3390/biom16101442)
Supplement: Supplementary file 1 [file biomolecules-16-01442-s001.zip › biomolecules-4356865-supplementary.pdf]

Supplementary Material

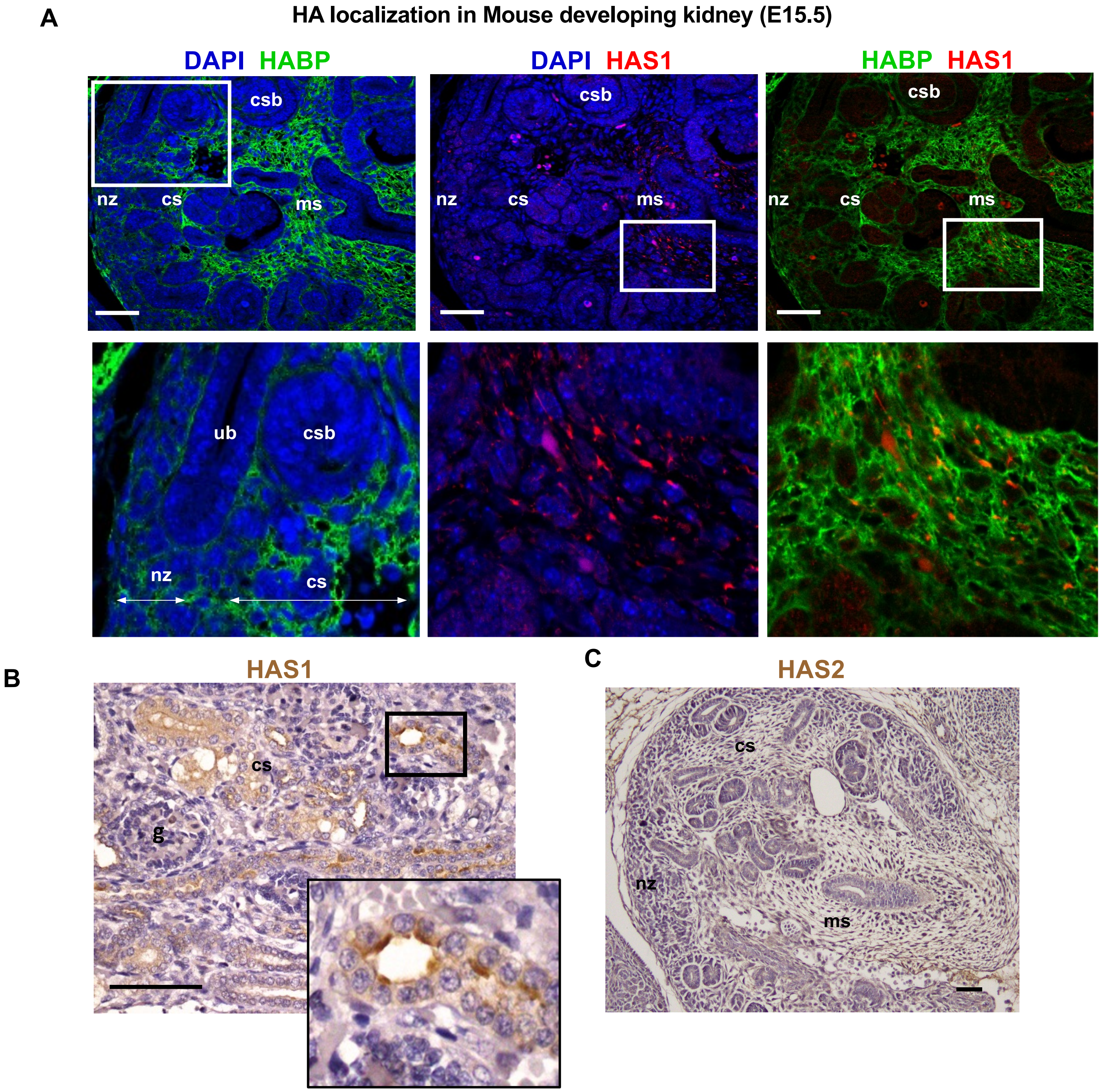

Supplementary Figure S1. **(A)** Immunofluorescence detection of HA binding protein (HABP, *green*) and HAS1 (*red*) in mouse embryonic day (E) 15.5 kidneys demonstrating abundance of HA and HAS1 in the stroma of the nephrogenic zone (nz) surrounding ureteric bud (ub) branches and induced nephrons in the cortical stroma (cs) grouping around sets of tubules, and in the stromal interstitium of the medulla. Boxed regions are depicted at higher magnification (*lower panel*) Scale bars 50 µm. Original Magnification x400. **(B-C)** Immunohistochemical detection of (B) HAS1 and (C) HAS2 demonstrating presence of HAS1 and absence of HAS2 in developing mouse kidneys at E15.5.

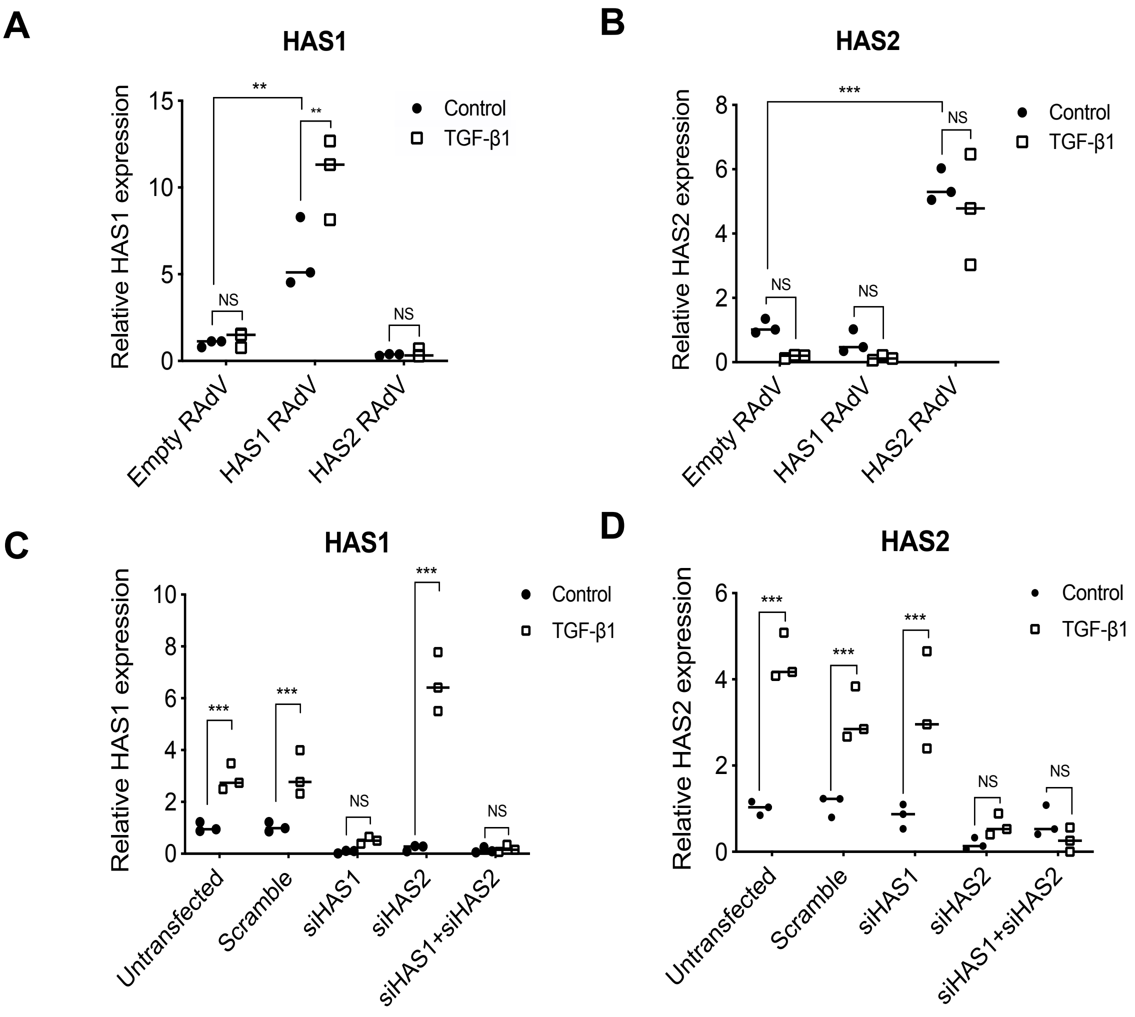

Supplementary Figure S2. Confirmation of *HAS* isoenzyme over-expression and knockdown. (A, B) Empty recombinant adenoviral vector (RAdV) and *HAS1* or *HAS2* RAdVs were used to infect oral mucosal fibroblasts in the absence (●) or presence (□) of 10 ng/ml TGF-β1 for 72 hours. RT-qPCR was used to assess mRNA expression of *HAS1* and *HAS2* to confirm over-expression. Dermal fibroblasts were transfected with scrambled siRNA or siRNA targeting *HAS1*, *HAS2* or both and incubated in the absence (●) or presence (□) of 10ng/ml TGF-β1 for 72 hours. RT-qPCR was used to assess mRNA expression of *HAS1* and *HAS2*. Data are presented as dot plots representative of 3 separate experiments. ***P*<0.01; ****P*<0.001. **(C, D)** Dermal fibroblasts were transfected with scrambled siRNA or siRNA targeting *HAS1*, *HAS2* or both and incubated in the absence or presence of 10ng/ml TGF-β1 for 72 hours. RT-qPCR was used to assess mRNA expression of *HAS1* and *HAS2.*

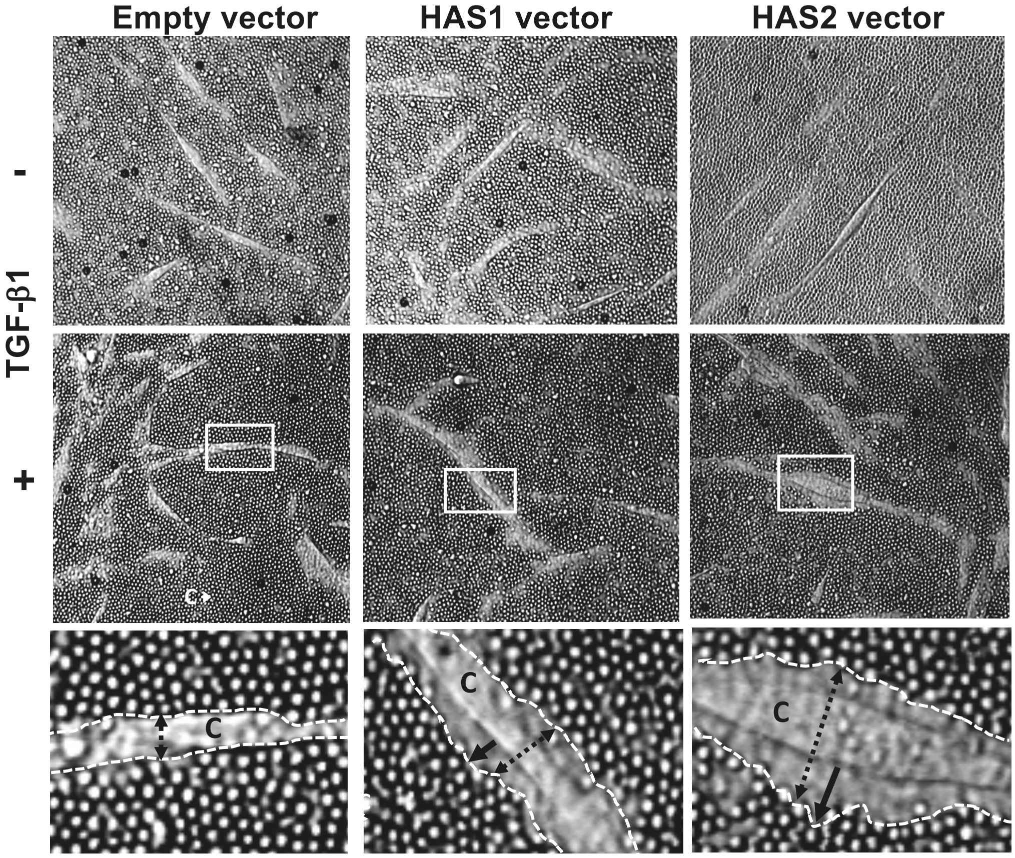

Supplementary Figure S3. Pericellular matrices HA were investigated using the red cell exclusion assay and observed using light microscopy. Higher magnification images of boxed regions are show below; dashed line indicates the widest part of cell analysed, and solid line depicts the measurement taken for pericellular coat quantification.

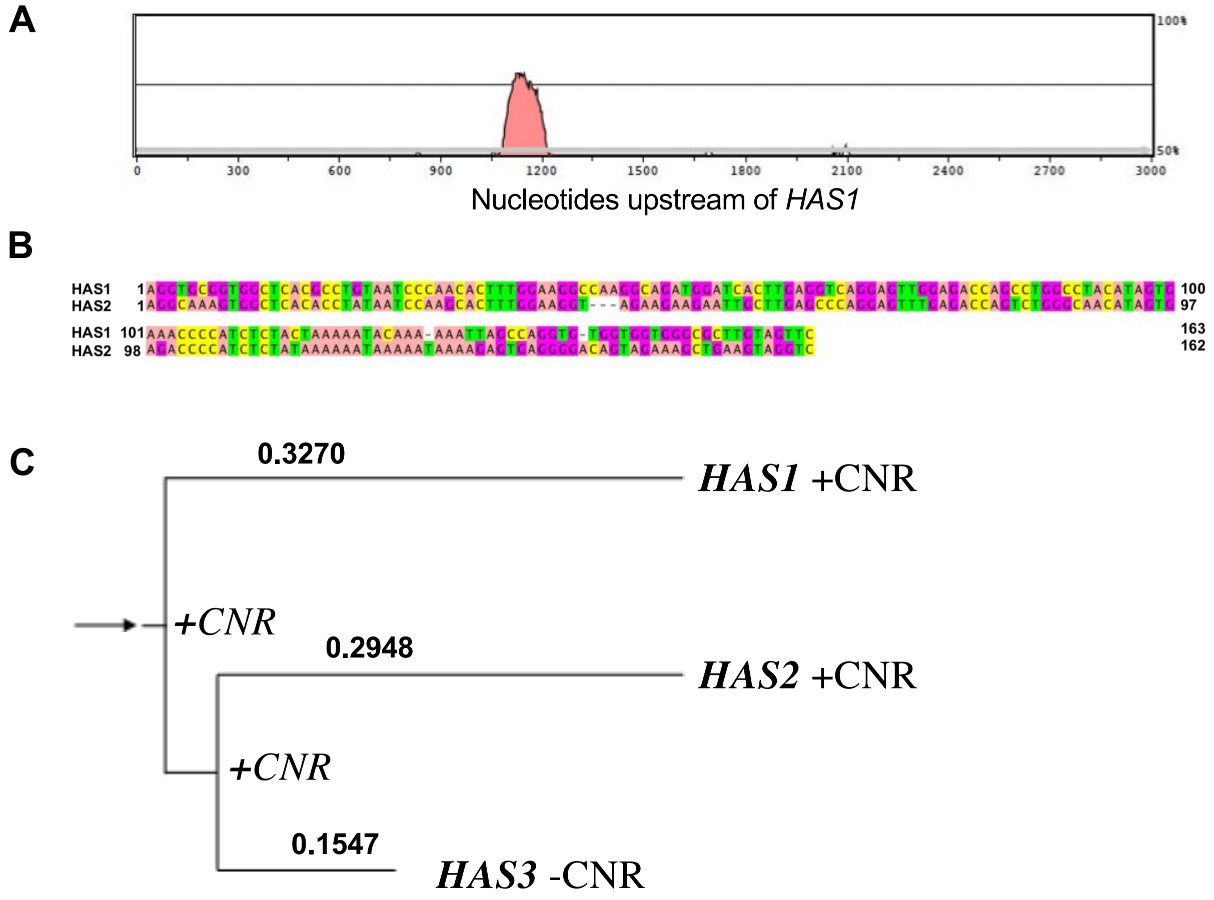

Supplementary Figure S4. Human *HAS* gene promoter sequence alignment and phylogenetic *HAS* analysis. (A) VISTA output for a comparison of 3 kb upstream regions from *HAS1* (transcript HAS1-001; Ensembl genomic coordinates Chromosome 19: 52,216,365-52,227,247 reverse strand) and *HAS2* (transcript HAS2-001; Chromosome 8: 122,624,356-122,653,630 reverse strand). Sequences were extracted from Ensembl release 70, which uses the GRCh37.p10 assembly. The red-shaded region represents a sequence >70% conserved between *HAS1* and *HAS2* and is shown with nucleotide positions relative to the *HAS1* transcription start site at 3001. **(B)** LAGAN alignment of the conserved region identified by VISTA, visualised using JALVIEW. **(C)** Phylogenetic analysis of human *HAS* gene cDNA sequences extracted from the Ensembl database. Sequences analysed were *HAS1-001* (ENST00000540069.2; labelled here as HAS1), *HAS2-001* (ENST00000303924.4; *HAS2*) and *HAS3-001* (ENST00000306560.1; *HAS3)*. Numbers on the branches are branch lengths (sequence differences) from the midpoint of the tree, which is marked with an arrow. The presence or absence of the CNR is indicated by "+CNR" for presence and "-CNR" for absence. Inferred presence of the CNR at branch points is indicated in italics.
